# Humeral metastasis as the only recurrence of a 5-year resected gastrointestinal stromal tumor: a case report

**DOI:** 10.1186/s13256-021-02962-8

**Published:** 2021-08-18

**Authors:** C. Braunstein, F. Sirveaux, E. Kalbacher, S. Aubry, D. Delroeux, P. Hubert, B. Marie, G. Meynard, I. Mihai, L. Chaigneau

**Affiliations:** 1 Service d’Anatomie Pathologique, Hôpital Nord Franche-Comté, Trévenans, France; 2 Service de Chirurgie Orthopédique, Centre Hospitalo-Universitaire de Nancy, Laxou, France; 3 Service d’Oncologie Médicale, Centre Hospitalo-Universitaire de Besançon, Besançon, France; 4 Service de Radiologie et d’imagerie Médicale, Centre Hospitalo-Universitaire de Besançon, Besançon, France; 5 Centre de Chirurgie Viscérale, Clinique St-Vincent, Besançon, France; 6 Service d’Oncologie Médicale, Centre Hospitalo-Universitaire de Besançon, Besançon, France; 7 Service d’Anatomie et Cytologie Pathologiques, Centre Hospitalo-Universitaire de Nancy, Laxou, France; 8 Service d’Oncologie Médicale, Centre Hospitalo-Universitaire de Besançon, Besançon, France; 9 Service d’Anatomie et Cytologie Pathologique, Hôpital Nord Franche-Comté, Trévenans, France; 10 Service d’Oncologie Médicale, Centre Hospitalo-Universitaire de Besançon, Besançon, France

**Keywords:** Jejunal GIST, Imatinib, Surgery, Humeral bone metastasis, Case report

## Abstract

**Introduction:**

Gastrointestinal stromal tumors represent the most frequently encountered primary mesenchymal tumors. Whereas the liver and the peritoneum are known to be the preferential metastasis sites, no therapeutic standard has yet been established for the management of bone metastases because of their very low incidence. We report a unique example of a single humerus metastasis of a jejunal gastrointestinal stromal tumor.

**Case presentation:**

We report the case of a 72-year-old European woman whose jejunal gastrointestinal stromal tumor was resected in 2013 and treated during the following 3 years with imatinib (400 mg daily). In 2018, she developed a single humeral bone lesion that was identified as a gastrointestinal stromal tumor metastasis. After 7 months of imatinib intake, reconstructive surgery was performed. Pathologists confirmed the satisfactory histological regression and assessed the complete tumor resection. The patient is still on imatinib maintenance therapy, with no recurrence reported so far. She fully recovered the upper limb function after following an appropriate rehabilitation program.

**Discussion:**

Current literature and published case reports indicate that bones are one of the rarest locations of gastrointestinal stromal tumor metastasis (about 1%), with occurrence mainly in the spine. Patients initially diagnosed with gastrointestinal stromal tumor of the small intestine and stomach are more likely to suffer from bone metastasis, compared with other gastrointestinal stromal tumor locations. The median overall survival rate is higher for patients with isolated bone metastasis compared with those having liver metastasis. Metastasis occurs on average 4 years after the primary, but it may take up to 20 years, emphasizing the need for long-term clinical and radiological monitoring. Although specific guidelines for such cases have not yet been established, we suggest that a multimodal concerted approach involving surgery or radiotherapy associated with tyrosine kinase inhibitor intake should be considered.

**Conclusion:**

Bones are one of the rarest locations of gastrointestinal stromal tumor metastasis. A multidisciplinary collaboration was set up to allow conservative surgery of our patient after several months of imatinib treatment. A year and a half later, the patient is still in complete remission. This specific case supports the concept of an intermediate stage between local and oligometastatic disease that should be managed with a curative aim, as much as possible.

## Introduction

Gastrointestinal stromal tumors (GIST) are the most common mesenchymal tumors of the gastrointestinal tract. The incidence rate varies from 15 to 30 cases per million people, and keeps increasing [1–3].

Interstitial cells of Cajal in myenteric plexus are known to be the precursors to these mesenchymal tumors. GIST can involve any location in the gastrointestinal tract but are most frequently encountered in the stomach and small intestine [2, 4, 5]. A percentage of GIST as high as 85% results from independent mutations involving the tyrosine kinase proteins receptors exon 9 or 11 of C-KIT (CD117) gene or exon 18 of platelet-derived growth factor receptor alpha (PDGFRα) gene. These alterations lead to proliferation, survival, and cellular growth resistance through constitutively activated signaling pathways [2, 5, 6].

Complete resection remains the best option for localized tumors. Health care guidelines and prognosis rapidly changed after tyrosine kinase inhibitors (TKI) were brought to the market.

With modern targeted therapy, patient overall survival has increased, and the course of GIST diseases keeps evolving, as documented in the literature, requiring constant adaptation of patient care and therapeutic follow-up [7]. Prevalent metastasis locations are in the liver and peritoneum [3, 7, 8]. However, bones and lung have also been found to be susceptible to host metastasis [8–13], but guidelines have not yet been established owing to their very low incidence.

We report the medical history of a 72-year-old woman who developed a single humeral bone metastasis 5 years after complete surgery of the primary tumor.

## Case presentation

The 72-year-old Mrs T. of European origin consulted her physician because of symptoms of abdominal distension, dyspepsia, and anorexia, which caused a 5 kg weight loss. Her personal medical history consisted of drug-induced chronic pancreatitis and a resected benign breast nodule. She stated that she had no allergies and no smoking habits. Chronic medication consisted of a daily pancreatic enzymatic substitution. The patient was professionally active as an employee in an insurance company, with no relevant exposure reported. Her family history included a case of colorectal cancer and of breast cancer, for an aunt and a cousin, respectively.

Radiological examinations reported a mobile pararectal mass at least 8 cm long, without any other lesion. Blood markers (Human Chorionic Gonadotropin, serum alpha feto-protein, lactate dehydrogenase, Carcino-embryonic antigen, tumoral antigen 19-9, and tumoral antigen 125) levels were in the usual range. Initial explorative celioscopy in August 2013 was subsequently converted to laparotomy, which allowed complete resection of a jejunal tumor. No other abdominal nor pelvic lesion was detected. Pathological analysis confirmed the diagnostic of a GIST with typical immunohistochemical profile and strongly positive DOG1, CD34, and CD117 markers. The Ki67 proliferation index was evaluated at 10%, and the necrosis contingent was below 50%. Molecular sequencing revealed that the exon 11 of C-KIT gene was deleted (as in 70% of GIST) [5].

Considering that the tumor was located in the intestine, that its largest dimension was 11 cm and its mitotic index higher than 5 per mm^2^, it was classified as belonging to the 6b group in the Miettinen classification [6], with an associated metastatic risk higher than 90%. Circumferential margin was evaluated null, and no serous breaking was observed. The latter mostly determines the local recurrence risk and the overall survival in intermediate or high-risk localized GIST [14]. Consequently, the oncology committee approved a 3-year adjuvant therapy with imatinib dosed at 400 mg per day, as recommended. The patient went through active controls every 4 months during the first 3 years and then every 6 months during the following 2 years after the imatinib treatment was completed. Active follow-up consisted of standardized quality of life evaluations, clinical examinations, and computed tomography scanners. Analysis of the liver and renal functions and blood count monitoring were also performed.

**Fig. 1:**
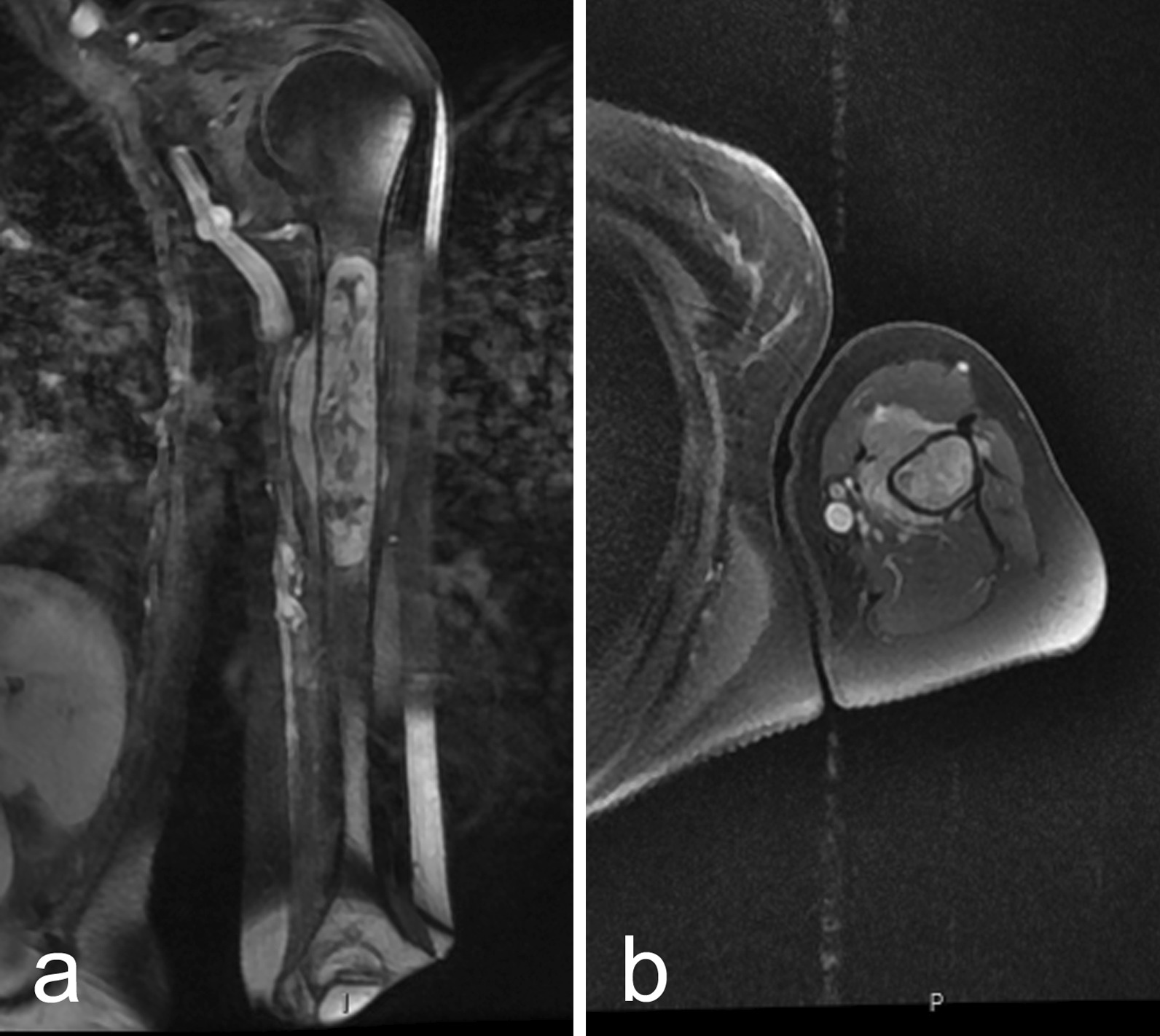
October 2018, magnetic resonance imaging (MRI) scan of the left humerus before treatment. Coronal (**a**) and axial (**b**) contrast-enhanced fat-suppressed T1-weighted images. Diaphyseal metastasis with extension to adjacent soft tissue highly enhanced

**Fig. 2:**
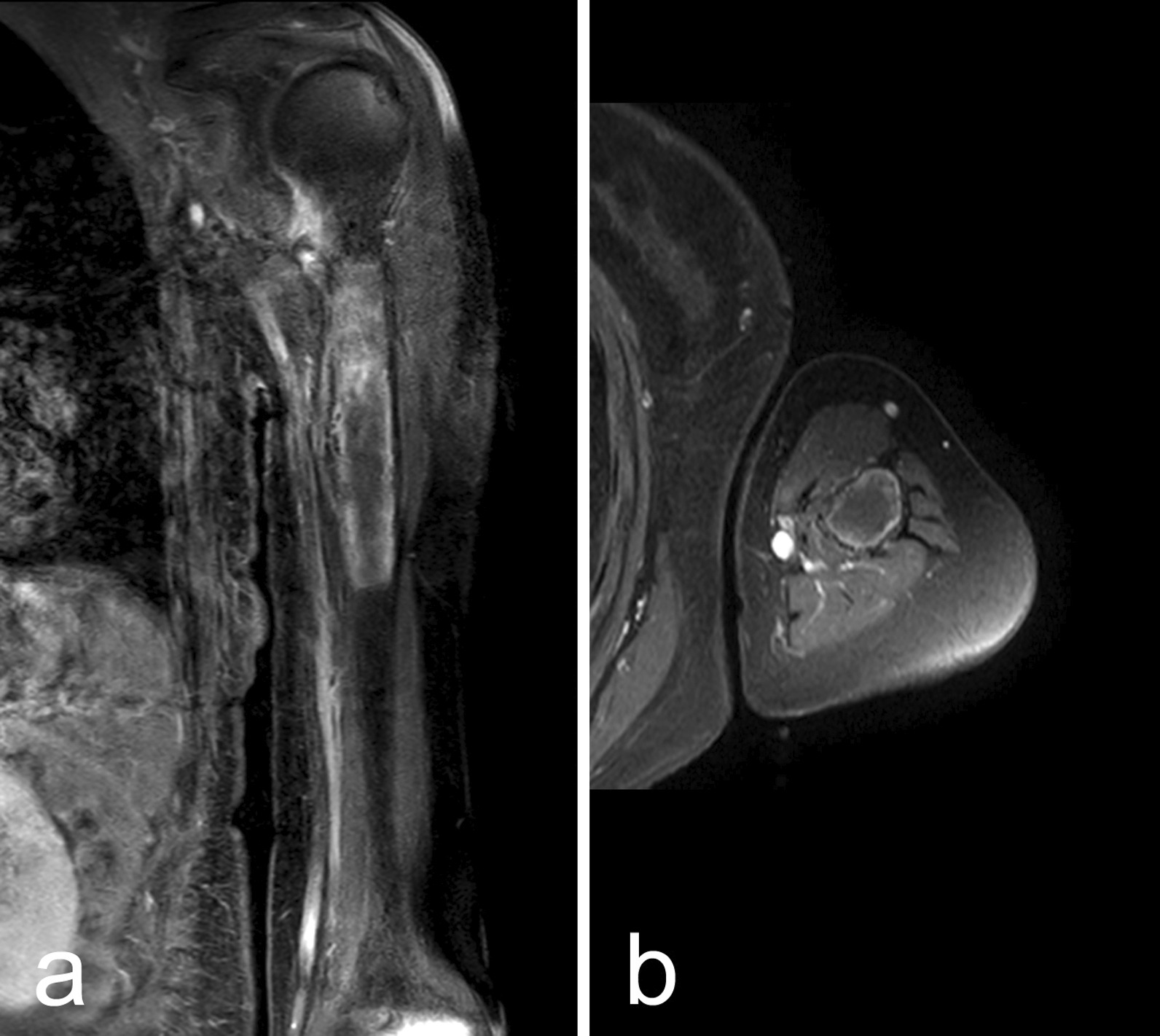
February 2019: MRI of the left humerus after 3 months imatinib treatment. Coronal (**a**) and axial (**b**) contrast-enhanced fat-suppressed T1-weighted images. See the central necrosis and residual peripheral enhancement

No clinical event happened until October 2018, when the patient reported shoulder and left-arm painful functional limitation. Humeral bone swelling was clinically palpable. Brachial magnetic resonance imaging (MRI) and computed tomography (CT) (Fig. 1a, b) evidenced a 10 cm-long heterogeneous tumor localized on the half-proximal left humeral bone. It was characterized by a low signal in T1-weighted pulse sequence and a heterogeneous intense signal in T2-weighted pulse sequence. Gadolinium injection revealed a heterogeneous contrast enhancement. Lesion borders were clearly delimited with a significant homogeneous contrast in proton-density-weighted signal, also after gadolinium injection. No significant cortical reaction or muscular invasion was observed. No other suspicious lesions were detected by body scan and bone scintigraphy. The analysis of percutaneous bone biopsies confirmed the diagnosis of a GIST. The oncology committee validated the reintroduction of imatinib dosed at 400 mg per day from mid-December 2018, and then approved surgical resection in view of the good clinical and radiological responses (Fig. 2a, b). Surgery performed in May 2019 consisted of a 15 cm humeral diaphyseal resection below the surgical neck and, at the same time, reconstruction with intercalary allograft. Fixation was achieved with locked nail and plate materials.

**Fig. 3:**
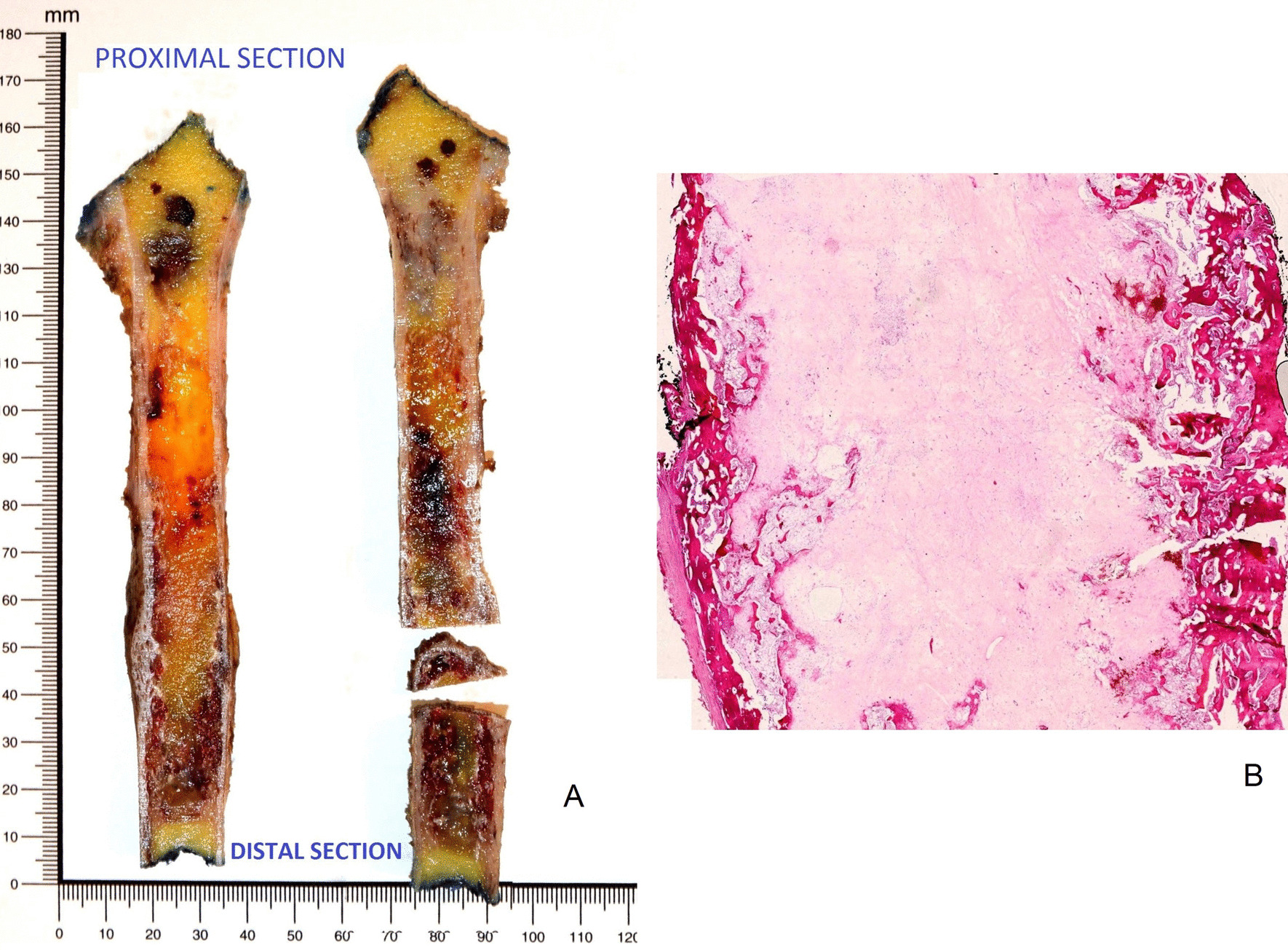
Macroscopic presentation after anteroposterior opening of the surgical humeral bone resection of 15.5 × 2.4 × 3.8 cm (**A**)**:** It measured 9 cm on height, and reached the proximal section at 3.5 cm and distal section at 0.8 cm. Note the reactional cortical hypertrophy associated. Histological presentation at low magnification (Hematoxylin-eosin stain (HE stain), ×0.5 magnification, **B** Tumoral area presented as a heterogeneous aspect including hemorrhagic and fibrous modifications

**Fig. 4:**
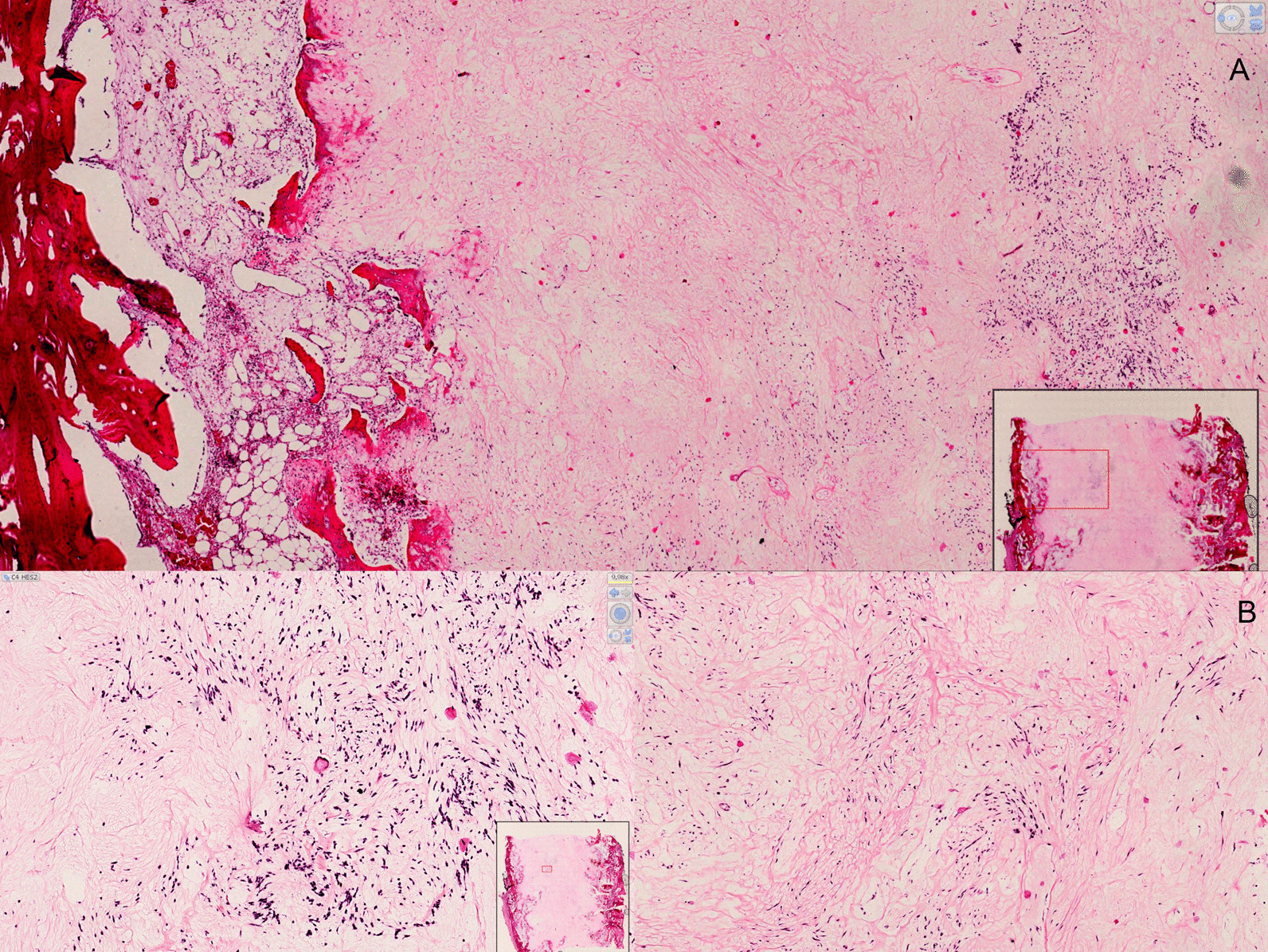
Histologic features: Hematoxylin-eosin stain (HE stain), ×2 (**A**) and ×10 magnification (**B**). Post-therapy regression presented as large fibrous cicatricial sheets containing focal areas of monomorphic spindle cells poorly atypical. Reduction and thickness of the cortical bone with reactional ossification were associated

**Fig. 5:**
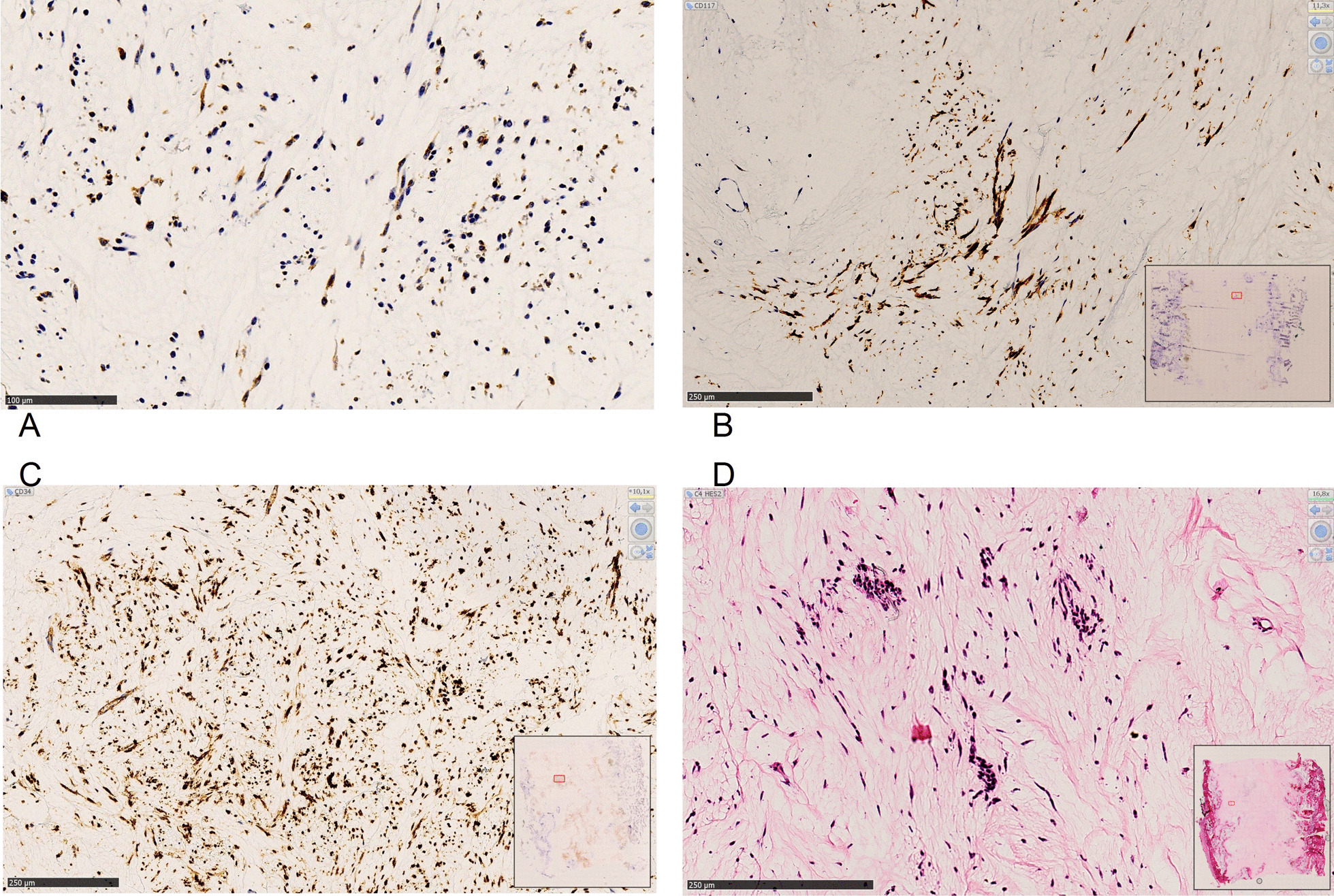
**A**–**D** Immunohistochemistry markers (×10 magnification): immunohistochemistry profile with DOG1 (**A**), CD117 (**B**), and CD34 (**C**) strong positivity combined with typical morphologic aspect of spindle cells (**D**) confirmed of a GIST.* Signal detection system EnVision FLEX + DAKO. Automate DAKO OMNIS*

Pathological analysis consisted of macroscopic examination after anteroposterior opening to select areas of interest for systematic sampling. The surgical humeral bone resection measured 15.5 × 2.4 × 3.8 cm. The tumor area of 9 cm in height had a heterogeneous aspect, including hemorrhagic and fibrous modifications (Fig. 3). A margin of 0.8 cm from the tumor to the distal section evidenced complete surgical resection. The significant histological regression consisted of poor contingent of spindle atypical cells and major fibrosis tissue reaction (Fig. 4). The immunohistochemical study with antibodies DOG1, CD117 (C-KIT), and CD34 confirmed the previous diagnosis of a GIST with a retained protein profile (Fig. 5).

**Fig. 6:**
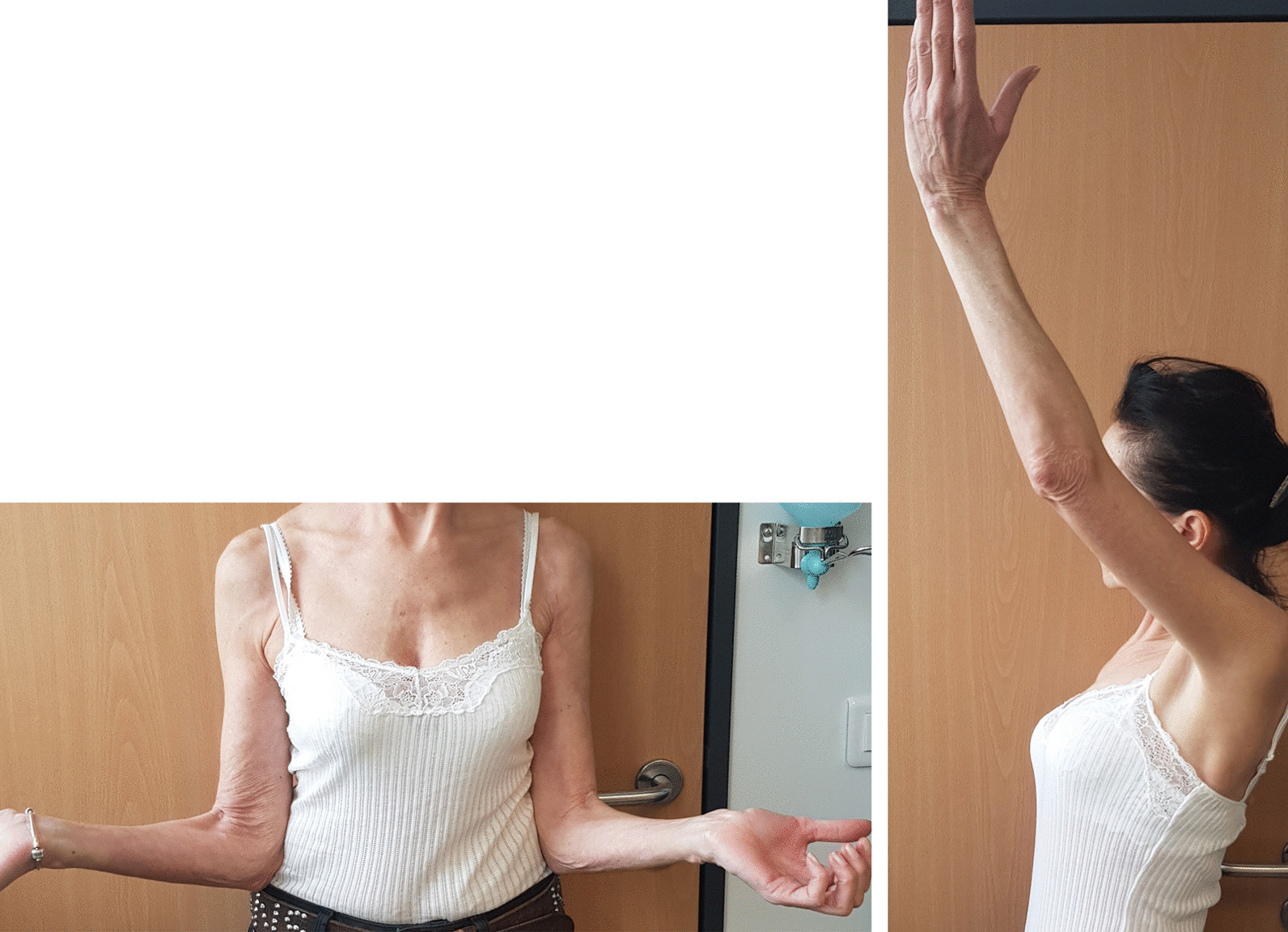
February 2020, postsurgery clinical presentation and functional examination. In June 2020, more than 1 year after the surgery, the patient fully recovered the upper limb function, the allograft is well integrated, and no recurrence is reported

**Fig. 7:**
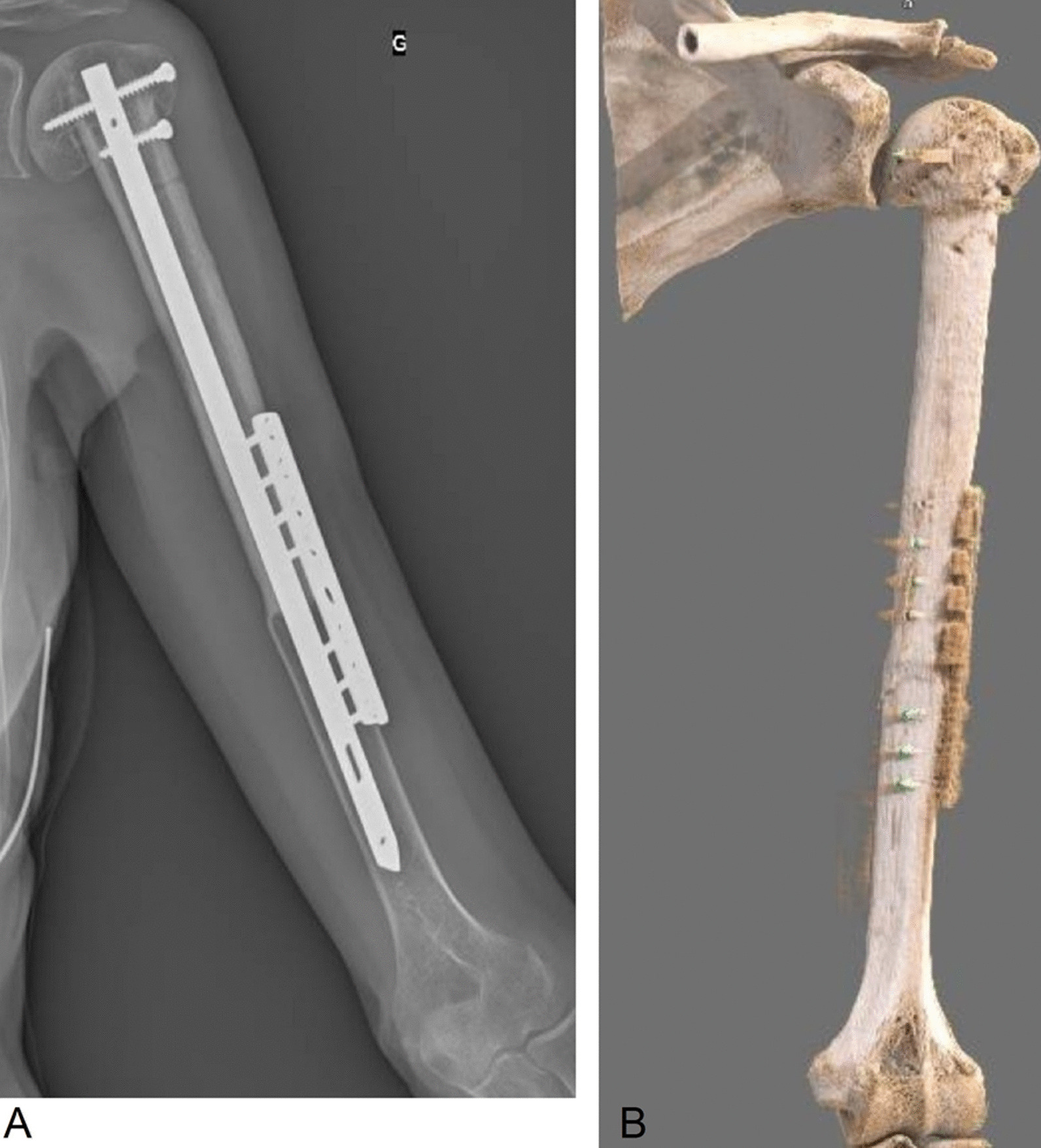
June 2020 examination with standard X-ray (**A**) and 3D-computerized tomography reconstruction (**B**)

Until now, the patient continues to take a daily dose of 400 mg imatinib, until disease progression or pharmacologic adverse events occur. In June 2020, more than 1 year after surgery, she fully recovered the upper limb function, the engraftment was successful, and no recurrence was observed (Figs. 6, 7).

## Discussion

Complete surgery is still the gold standard for localized GIST. Pathologic examination is important to confirm diagnosis and establish drug options and subsequent prognosis. Different risk classifications are commonly used. On the one hand, the American Force Institute of Pathology (AFIP) classification and the Miettinen classification are based on tumor size, mitotic count, and location [6]. On the other hand, the National Health Institute (NIH) established a mapping classification considering size, mitotic count, gastric or extragastric location, and tumor breaking [4, 5, 15].

Whereas bones and lung have also been found to be susceptible to host metastasis [8–13], no management consensus has been established owing to their very low incidence. These rare locations are most frequently associated with liver metastasis [1, 2], and the latter alter the median overall survival.

On the basis of literature data, the percentage of patients developing bone metastasis varies from 0.47 to 5.5% [3], with spine as the predominant bony site. Other bone metastasis locations are rare, with an occurrence of approximately 1% [9, 10, 13]. Small intestine and stomach tumor locations lead to more frequent and earlier bone metastasis compared with other sites.

Metastasis occur on average 4 years after diagnosis of the primary GIST, but can take up to 20 years to occur [8]. Thus, our report highlights the importance of long-term clinical and radiological patient monitoring, even after early diagnosis. Modalities of the necessary active controls (frequency, MRI or CT imaging, blood analysis) depend on initial recurrence risk. The duration of the surveillance remains debated. Medical oncologists must be aware of that and should not forget the possible occurrence of rare metastatic evolutions (bone, lung). Moreover, adjuvant therapy duration of imatinib after resection of the primary should perhaps be extended, considering the long-time onset, as the clinical trial ImadGIST aims to investigate [16].

We proposed a complete resection of the humeral metastasis, framed by a targeted therapy with imatinib, although standards of care recommendations are not yet established, because such cases have only rarely been reported [8]. After humeral bone metastasis diagnosis and preoperative treatment, no other lesion developed, making the surgery feasible and resulting in complete remission until now. The French sarcoma group studied how local ablative treatments increased the overall survival in oligometastatic bone and soft-tissue sarcomas [17].

Moreover, the development of well-known hepatic metastasis during evolution of colorectal cancer requires, for the patients considered eligible, an aggressive oncological and surgical approach to improve overall survival [18, 19]. These results support the concept of an intermediate stage between local and diffuse metastatic disease that should be managed more aggressively than standard palliative care. Targeted metastatic surgery, stereotaxic radiotherapy, or radiofrequency must be considered in addition to tyrosine kinase inhibitors treatments, depending on the molecules previously administrated. Imatinib, sunitinib, and regorafenib were proved effective in first, second, and third line of advanced or metastatic disease, respectively.

Recently, other TKI such as avapritinib or ripretinib appear to be interesting for fourth-line therapy or in cases of specific secondary resistance mutations [20, 21]. It can be concluded that multidisciplinary approaches are required to significantly increase the overall survival with a satisfactory quality of life [9, 10]. Current literature concerning solely bone metastasis or other metastatic sites highlights the precautions and dialogues needed to optimize the sequence of surgery modalities, radiotherapy, and oral drugs, for the benefit of the patient. Biological aspects require further investigations to understand the molecular pathways involved in tumor escaping. The problematic of heterogeneity of secondary resistance mutations has already been pointed out in 2006 [22, 23]. Two-thirds of the GIST cases develop secondary resistance mutations, affecting mostly the same allele as the first mutation detected, which result in therapy resistance [24].

No specific molecular profile among those identified [25] appears to be associated with aggressivity [3, 11]. Consequently, no recommendation for a systematic screening has been settled.

## Conclusion

Our knowledge of the evolution of diseases such as GIST is progressing, as witnessed in the current literature, and thus the need for medical oncologists to implement long-term patient surveillance, with modalities depending on the initial recurrence risk [7]. This report highlights the potential occurrence of delayed metastasis at least 5 years after the patient was diagnosed with a jejunal GIST. We draw attention to the benefits of a concerted, multimodal therapeutic approach taking into account the metastasis sites and the patient medical operability. State-of-the-art therapies offer targeted metastatic surgery and implementation of radiofrequency, or of stereotaxic radiotherapy, complemented with specific tyrosine kinase inhibitors, that have not yet been administered.

In conclusion, appropriate clinical and surgical management of such clinical cases requires active multidisciplinary collaboration between medical oncologists, radiotherapists, surgeons, and pathologists to achieve optimal overall survival and patient quality of life.

## Data Availability

The datasets used and/or analyzed during the current study are available from the corresponding author on reasonable request.

## Abbreviations

CT: Computerized tomography
GIST: Gastrointestinal stromal tumor
HE: Hematoxylin-eosin stain
MRI: Magnetic resonance imaging
TKI: Tyrosine kinase inhibitor
